# New Dimension on Quality of Life Differences among Older Adults: A Comparative Analysis of Digital Consumption in Urban and Rural Areas of China

**DOI:** 10.3390/ijerph192215203

**Published:** 2022-11-17

**Authors:** Zhizheng Zhang, Wentao Wei, Tianlu Zhu, Ming Zhou, Yajun Li

**Affiliations:** School of Design Arts and Media, Nanjing University of Science and Technology, Nanjing 210094, China

**Keywords:** digital consumption, quality of life, older adults, urban and rural differences, COVID-19 pandemic

## Abstract

The purpose of this study was to compare the variability in quality of life in the area of digital consumption among older adults in urban and rural China during the COVID-19 pandemic. This study proposed a low-cost mixed research method, and the methodology used a quantitative study of a large regional sample combined with a qualitative study of a small regional sample. Data for the large-scale area sample were obtained from the China family panel study (CFPS) dataset, and data for the small-scale regional sample were obtained from Nanjing, China. The quantitative analysis of the large-scale regional sample used the least squares regression analysis (OSL) and propensity score matching (PSM). The qualitative analysis of the small-scale regional sample used the selection optimization and compensation (SOC) model. The findings show that economic income is a direct driver of digital consumption. Digital consumption had a significant positive relationship with the quality of life for urban and rural older adults. In addition, the study established the semantic network relationships of the coping strategies of digital consumption of older adults and their drivers. Finally, the theoretical and practical implications of these findings are discussed in the context of other related studies.

## 1. Introduction

Throughout history, along with the evolution of social forms, economic development has passed through the agricultural and industrial eras. Nowadays, we are in the age of the information economy, with the integration, sharing, and increasing use of information technology in the digital economy. Especially during the COVID-19 pandemic, information and communication technology (ICT) has helped people establish new ways of living and consuming. Our grandparents experienced a shift from traditional consumption to digital consumption. Can their lives adapt to digital consumption? For the population quality of life survey, in the face of a large aging population and unbalanced development between urban and rural areas, it is particularly important to use a low-cost and efficient and accurate survey method. In particular, there is a need to consider the differences in culture, race, and faith in different countries. In this study, an empirical study was conducted on the elderly population in the Yangtze River Delta region of China. The study included a differentiated study on the quality of life for urban and rural older adults affected by digital consumption in a large-scale regional sample, and a study on the quality of life coping strategies for urban and rural older adults in a small-scale regional sample during the COVID-19 pandemic.

Currently, ICT services have deeply changed people’s life patterns. The advantages of quickly communicating and instantly sharing information have brought great convenience to life services [1]. At the same time, various countries and regions are continuously promoting the digitalization of services and consumption. However, there seem to be two different types of opinions appearing among older adults. One group of opinion considers that ICT services have a positive impact on the quality of life for older adults. For example, it helps the elderly to monitor their physical state and improves the quality of their health [2]. The quality of life is enhanced by promoting mental health and active social integration [3]. The other opinion is that ICT services have a negative impact on the quality of life for older adults. For example, the digital divide causes cognitive load and reduces life satisfaction [4,5]. The information cocoon effect reduces the information discrimination and social participation of older adults [6]. The difference between the two types of opinions is due to the difference between objective evaluation and subjective feedback. The representative behavior in ICT services is digital consumption, and consumption is not only an external behavioral expression, but also an internal psychological behavior. Therefore, in the study of the factors influencing digital consumption on the quality of life for older adults, not only should the objective indicators for quality of life be evaluated, but also the feedback on the subjective indicators of older adults should be considered.

In countries with large geographic areas and large populations, a large-scale survey of the quality of life for the population would be very costly in terms of time and money [7,8]. For example, each census round in China takes three years. Moreover, the elderly population is one of the most diverse groups in the population with specific regional and cultural differences [9]. The trend of ICT services represented by digital consumption factors is changing very rapidly, and the traditional high-volume survey method obviously cannot adapt to the needs of the digital consumption economy. To solve such problems, this study proposed a quality of life survey method of large-scale sample screening with small-scale sample depth, which combines the pattern of large-scale samples over long time spans with the characteristics of small-scale samples over short time spans for in-depth research on the basis of low cost. It is not only able to summarize the broad patterns from large samples, but also to obtain the regional demographic characteristics from small samples. Therefore, this study used the data of the Yangtze River Delta region in the China Family Panel Studies 2020 database (CFPS) as a large-scale sample and the survey data from the Nanjing area, which is part of the Yangtze River Delta region, as a small-scale sample.

According to available studies, the proportion for quality of life studies on chronic noncommunicable diseases (CNCD) in the elderly population is more than 35%, covering topics such as cardiovascular disease, diabetes, hypertension, dyslipidemia, and obesity [10]. It can be seen that the current studies on quality of life are focused on the field of physiological functions, and the instruments used to evaluate the level of quality of life are mainly various scales such as the Medical Outcomes Study Short-Form 36 (SF-36) [11], 12-Item Short-Form Health Survey (SF-12) [12], EuroQol (EQ-5D) [13], and Visual Analogue Scale (EQ-VAS) [14]. Among them, SF-12 is widely used in regions in China for quality of life survey studies. Therefore, SF-12 was selected as the evaluation tool for a large regional sample for the objective evaluation on the quality of life among older adults.

Although the use of the SF-12 evaluation tool will help to analyze the variability of older adults, it may hinder comparisons between samples with similar characteristics or needs, thus affecting the quality of the data analysis results [15]. To remedy this deficiency, the Selective Optimization with Compensation (SOC) model was added as an evaluation method for small regional samples in a subjective evaluation study on the quality of life among older adults. The model provides an explanation for successful aging in which people adapt to the loss of capacity in a way that optimizes their residual capacity and resources [16]. That is, older age groups optimize their weakened capacity during a pandemic in the form of digital consumption and redeploy resources around them to survive the pandemic crisis, thereby improving their quality of life.

Over the past decade, most studies on the quality of life for older adults in China have addressed self-rated health, daily activities, mental illness, and social intergenerational [17,18,19,20], however, there is no detailed geospatial delineation of older adults’ living areas in these studies. As a result of urbanization in China, the movement of laborers into the cities has led to a situation in which a large number of elderly people live alone in rural areas. There is an objective disparity in the level of development between urban and rural areas. Less developed facilities and social services may result in older adults being more vulnerable to the negative effects of the pandemic. To fill in the factors influencing the living areas in the study on the quality of life for older adults in China, this study discusses the different characteristics of urban and rural elderly populations affected by digital consumption, which can help to narrow the gap in quality of life for populations in different regions. Establishing targeted digital transformation policies and services can compensate for the adverse geospatial impact of the pandemic. This is a new dimension and opportunity to improve the quality of life for older adults in urban and rural areas.

**Figure 1:**
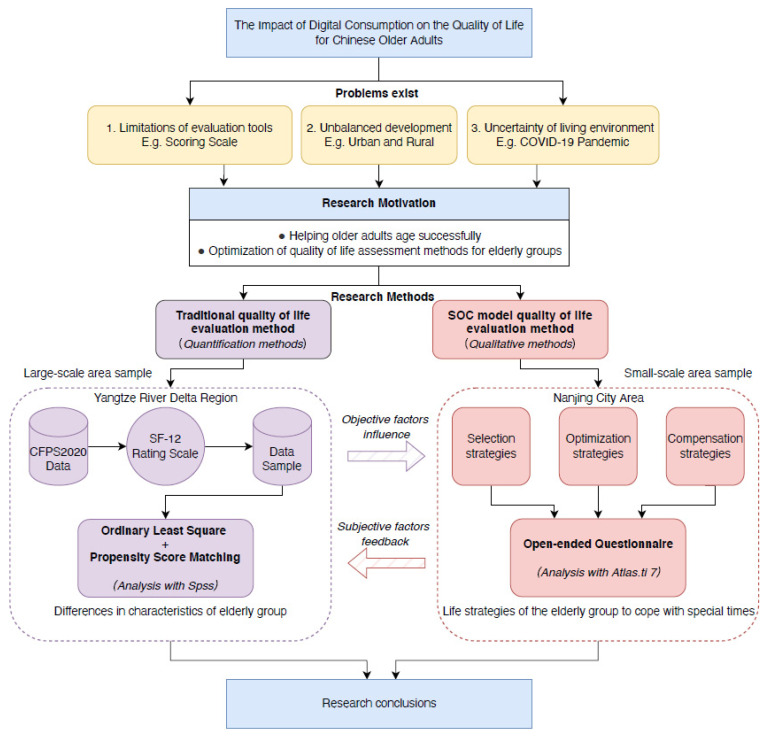
The study flow diagram. Note: SOC—selective optimization with compensation.

As shown in Figure 1, the innovations and contributions of this study are reflected in the following points. In terms of research questions, this study focused on the effect of digital consumption on the quality of life for older adults and the differential relationship between urban and rural areas, an issue on which few researchers have focused. In terms of research design and methodology, this study will use ordinary least square regression analysis (OSL) and propensity score matching (PSM) methods to more accurately analyze the objective impact effects of digital consumption on the quality of life for older adults. The SOC theory model was used to evaluate the subjective impact on the elderly group, and the extended application of SOC theory in the field of digital consumption, and quality of life was explored to further explain the quality of life gap between the urban and rural elderly groups.

The rest of this paper is structured as follows. Section 2 includes the literature review and research hypotheses. Section 3 presents the research methods and materials as well as the data collection and data analysis. Additionally, the results of the empirical analysis are presented in Section 4. Finally, Section 5 presents a discussion of the study and its limitations, and Section 6 provides a summary of the conclusions.

## 2. Literature Review

### 2.1. Digital Consumption

Digital consumption, as a specific behavioral manifestation of participation in information and communication technology (ICT) services, satisfies the acquisition of material resources on one hand and compensates for the dependence on psychological resources on the other [21]. However, due to public service place closures in times of public health crisis, more and more goods and services are moving away from offline entities and transforming into online virtual services. During a difficult pandemic, older adults have been actively or passively exposed to digital consumption patterns. While the impact of digital consumption on older adults may be double-edged, it is undeniable that digital consumption patterns will be the mainstream form of consumption in the future and will deeply impact the lives of older adults.

First, digital consumption makes consumer behavior traceable by improving resource utilization and serving more needs through information and sharing [22]. Second, consumer behavior is influenced by cultural, environmental, and national differences [23]. Studies have shown that older users in different countries show differences in their behavior toward traditional versus digital media consumption [24]. Meanwhile, the potential positive and negative impacts of digital consumption in different contexts cannot be ignored. For example, Kanungo et al. [25] mitigated the effects of digital harm caused by excessive digital consumption through the lens of two complementary theories. Frick et al. [26] examined how perceptions of the online environment affected the individuals’ levels of consumption of travel, apparel, and digital equipment. It can be seen that digital consumption is multifaceted, with different characteristic variables leading to variability. It can be seen that the composition of digital consumption is multidimensional and needs to consider the community services and consumption habits for older adults.

### 2.2. Community Services and Consumption Habits

Compared to Asian countries such as Japan, Korea, and India, China is a country that places the most emphasis on traditional family values and has vigorously promoted the community service centered aging in place model since the population has been aging [27]. Therefore, the elderly cannot live and consume without the system of community services. In the existing studies on community services for the elderly, the research content has mainly been discussed around the needs of community services for the elderly. For example, Duppen Rn et al. [28] studied the loss of social meaning among socially vulnerable older adults around social needs. Goodman-Casanova et al. [29] studied the health and well-being of community-dwelling older adults with mild cognitive impairment based on television and telephone health needs and social support. Ko et al. [30] examined gender differences in the community service needs of older adults in seven areas: housekeeping needs, facility needs, environmental needs, exercise needs, family needs, emotional needs, and ambulance needs. By sorting through existing community service studies, the following six categories of community service needs were summarized: life care needs, medical and health needs, travel needs, social support needs, education needs, and social participation needs.

**Figure 2:**
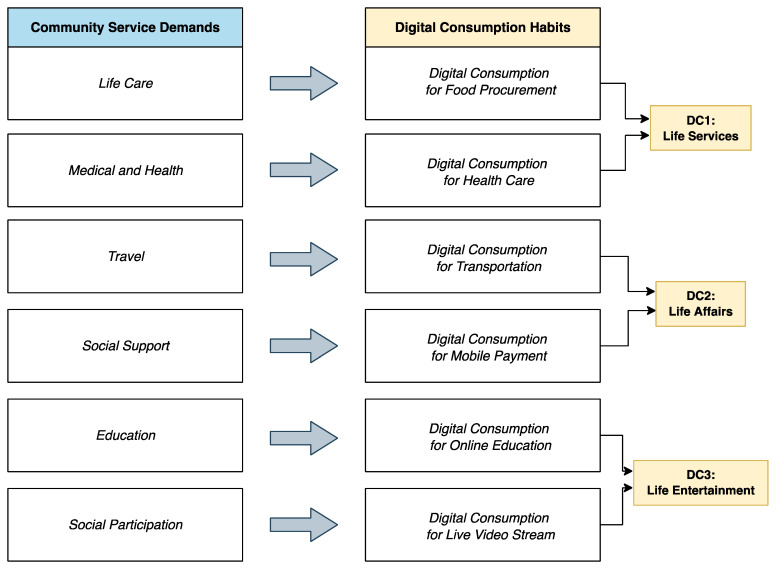
Relationship between the community service demand and digital consumption habits.

Changes in community services will change people’s consumption habits, as verified in Husain and Ashkanani’s study on the impact of changes in community activities during COVID-19 [31]. Therefore, as shown in Figure 2, mapping the relationship between the above six categories of community service needs and digital consumption habits was established to construct three categories of the behavior dimensions of digital consumption. DC1 to DC3 will be used as explanatory variables in the follow-up data analysis.

### 2.3. Quality of Life for Older Adults

So far, the literature lacks a clear definition of the concept of quality of life. It has been stated that quality of life is the degree to which a person is satisfied with his or her living conditions [32,33]. Furthermore, quality of life is composed of multidimensional factors including economic factors (such as income, livelihood security, and insurance), social factors (such as interpersonal relationships, family, health, and social security), political factors (such as regional governance, government institutions, public participation, public services, and public credibility), and environmental factors (such as environmental quality, transportation and natural conditions) [34,35]. The current research objects related to the quality of life of elderly people mainly focus on physiological indicators. The number of studies on the evaluation of quality of life dominated by digital consumption behavior is still relatively small. The six behavioral dimensions of digital consumption can correspond well to the evaluation indicators for quality of life.

This study, based on the SF-12 evaluation indicators, will discuss two dimensions for quality of life, which consists of a physical component summary (PCS) and mental component summary (MCS). The PCS measures the general health (GH), physical function (PF), role physical (RP), and body pain (BP). The MCS measures vitality (VT), social function (SF), role emotion (RE), and mental health (MH). The PCS and MCS will be used as predicted variables in the follow-up data analysis.

Moreover, as Saha et al. [36] mentioned in their study on a framework that helps gauge older adults’ quality of life, East, South Asian, and Southeast Asian countries have different traditional ideas about aging compared to Western countries, and study on the quality of life for older age groups is still a relatively small field in China. In particular, unlike Western older adults who promote independence, Chinese older adults rely on state policies and are influenced by Confucianism. This reflects the difference between Chinese and Western ideologies and culture, and the existing studies on the quality of life evaluation models are mainly based on the American and European contexts. The different cultural backgrounds and social structures related to aging have led to the poor applicability of the existing evaluation models to older age groups in regions such as East Asia. Therefore, in this study, we used a combination of a quantitative study with a large sample and a qualitative study with a small sample in order to evaluate the quality of life of Chinese older adults more accurately at low cost, and to minimize the evaluation errors caused by regional cultural differences.

### 2.4. Impact of Pandemic on Quality of Life for Older Adults

During the pre-pandemic period, many older adults reported feeling lonelier and more isolated than they normally would. Several studies have shown that this trend continued during the pandemic [37,38,39]. In contrast to the isolation experienced by older adults at the psychological level, the physical level was limited by the closure of public places and the temporary closure of areas. Older adults and particularly vulnerable groups became more dependent on the rest of society for help and support during the pandemic [40]. The massive deployment of information technology and digital development during the pandemic has given more older adults access to ICT [41]. 

We have categorized the studies on quality of life for older adults during the two-year period of the current pandemic in both positive and negative terms, and the main findings of these studies are presented in Table 1. There have been numerous negative effects on the quality of life of older adults after COVID-19. For example, after investigating the pandemic in those aged over 60 years in Canada, the researchers discovered a decline in their life quality, health, and well-being [42]. During the Italian pandemic, the quality of life of the population declined due to the lack of adequate public health services [43]. At the same time, there were positive effects such as stimulating an increase in home-based skills education and reducing psychological stress among older adults during the COVID-19 pandemic [44]. In contrast, older adults in religious areas exhibited high psychological resilience during the pandemic, enhancing quality of life by tolerating stress and suffering [45]. According to the aforementioned studies, the quality of life of older adults during a pandemic is easily influenced by cultural context, region, and policy. We attempted to propose three control variables based on life course stage theory [46], namely, economic income, social relationships, and life circumstances.

### 2.5. Selective Optimization with Compensation Model

Both the selective optimization with compensation (SOC) model and quality of life are methods for assessing the physical and psychological factors in older adults. The classical theory of SOC, proposed by Baltes [51], provides an explanation of successful aging by explaining that older adults reallocate resources through selection, optimization, and compensation in order to achieve successful aging. Current research on the SOC model focuses on aging, illness, and social support. For example, Zhang and Radhakrishnan [52] examined the relationship between chronic illness and life satisfaction. Müller et al. [53] examined the relationship between aging and work balance among nurses in Singapore. Yates et al. [54] examined the development of a Promoting Independence in Dementia intervention to enhance independence in people with dementia.

For the assessment of quality of life in large samples, data analysis can only reflect the objective patterns. The SOC model provides a systematic way to observe and analyze the subjective feedback of older adults and the process of successful aging, and the SOC model can help to specify appropriate services for policy by explaining it to a small sample.

## 3. Materials and Methods

To achieve the study objectives, this study consisted of a quantitative regional study with a large sample size and a qualitative study with a small sample size. The data for the large sample were obtained from the China Family Panel Study 2020 (CFPS), which is an open access demographic survey database. The survey data in CFPS contain data on a variety of topics including social, economic, demographic, educational, and health changes in China, and fully meets the sample requirements for this study. The data for the small sample were obtained from an offline field survey conducted by this research team, and all respondents were aware of and signed an informant statement. This section contains a detailed description of the study design, respondents, study site, measurement, data collection, data variables, and data analysis for both studies.

### 3.1. Quantitative Study of Large-Scale Sample

#### 3.1.1. Study Design

**Figure 3:**
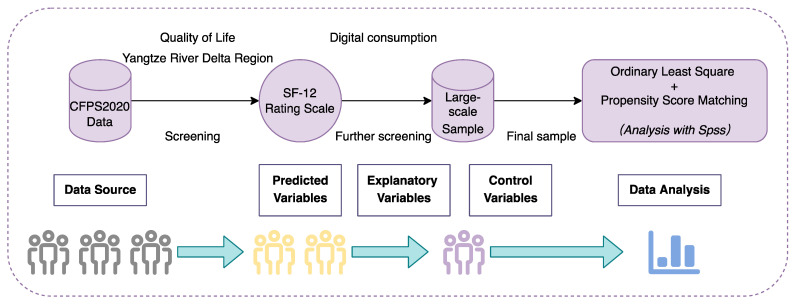
Quantitative study design.

The quantitative study design for the large-scale sample is shown in Figure 3. We screened the data in CFPS 2020 with reference to the indicators in the SF-12 evaluation scale [55], and the following indicators were screened: general health (GH), physical function (PF), role physical (RP), body pain (BP), vitality (VT), social function (SF), role emotion (RE), and mental health (MH). After establishing the preliminary data sample, we restricted the regions of data sources according to the needs of the digital consumption study and finalized the data sample for the quantitative study.

#### 3.1.2. Respondents and Study Site

**Figure 4:**
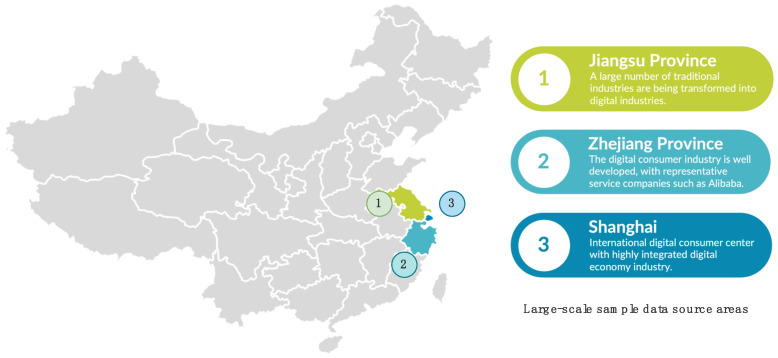
Location of the Yangtze River Delta region.

The CFPS 2020 data cover 14,960 urban and rural households in 635 villages across 162 counties in 25 provinces in China. The selected sample of older adults ranged in age from 60 to 80 years. The Yangtze River Delta region was chosen as the study site due to the rapid development of the digital economy in the Yangtze River Delta region of China in the last decade and the availability of a mature service system in the region. As shown in Figure 4, the geographical composition of the region consists mainly of Jiangsu Province, Zhejiang Province, and Shanghai.

#### 3.1.3. Measurement

(1) Dependent variables

The dependent variable was the quality of life (QoL), as measured by their performance on two scores: the physical component summary (PCS) and the mental component summary (MCS). The PCS was composed by GH, PF, RP, and BP. The MCS was composed by VT, SF, RE, and MH. Variables were measured using a five-point scale. Table 2 shows the criteria for the measurements.

(2) Independent variables

The independent variable was digital consumption (DC), which consists of digital life services (DC1), digital life affairs (DC2), and digital life entertainment (DC3). The variables were measured using a dichotomous approach, and the criteria for the measurements are shown in Table 3.

(3) Control variables

The control variables were economic income, social relationships, and living environment. Economic income consisted of the pension (PO) and financial support from children (FS). Social relationships consisted of group activities (GA) and interpersonal relations (IR). Living environment consisted of care support (CS) and facility convenience (FC). The variables were measured using a dichotomous approach, and the criteria for the measurements are shown in Table 4.

#### 3.1.4. Data Collection

The CPFS 2020 survey was conducted over a two-year period and was collected using face-to-face interviews and self-administered surveys. A valid sample of 1276 was selected from the CPFS 2020 dataset for the Yangtze River Delta region. Among them, 660 were male and 616 were female. A total of 1056 were from urban samples and 220 from rural samples.

#### 3.1.5. Data Analysis

The variables of digital consumption and quality of life in this study involved economics and sociology. The areas in which SPSS specializes include social sciences and economics [56]. Therefore, SPSS 25.0 version for Mac (IBM, Armonk, NY, USA) was used for data analysis. Ordinary least square regression analysis (OLS) and propensity score matching (PSM) were used to analyze the explanatory variable “digital consumption” and the predicted variable “quality of life”.

### 3.2. Qualitative Study with Small-Scale Sample

#### 3.2.1. Study Design

**Figure 5:**
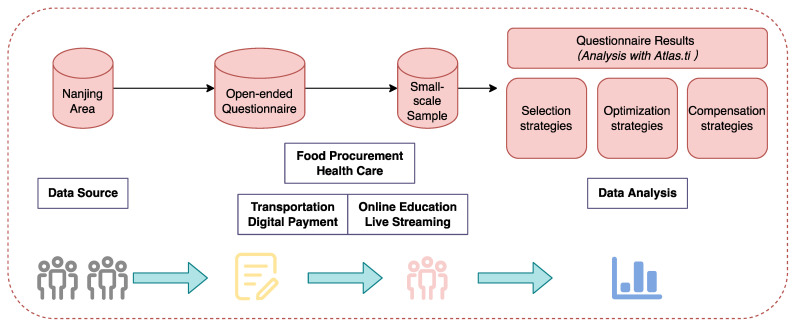
Qualitative study design.

The qualitative research design for a small-scale sample size is shown in Figure 5. We designed an open-ended questionnaire research method, which is more conducive to obtaining the respondents’ subjective feedback than focus groups [57]. The specific questions in the questionnaire were developed based on three categories of variables of digital consumption. A total of six subjective questions corresponded to DC1, DC2, and DC3. 

#### 3.2.2. Respondents and Study Site

**Figure 6:**
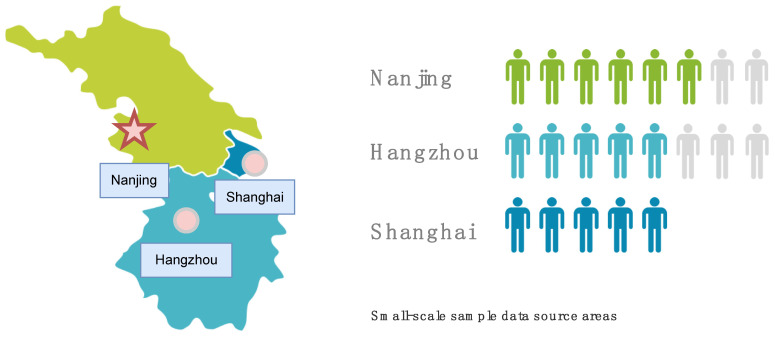
Location of Nanjing.

The elderly participants were between 60 and 80 years of age. Nanjing was chosen as the study site because it is located in the Yangtze River Delta region and is the capital city of Jiangsu Province for small sample collection. Nanjing covers a rich range of digital economy services and has a more concentrated elderly population than Hangzhou and Shanghai. These conditions are well-suited for small-scale sample collection (Figure 6).

#### 3.2.3. Measurements

In the qualitative study with a small sample size, as shown in Table 5, the questionnaire collected six separate items of subjective feedback on digital consumption behavior, for example, food procurement, health care, transportation, digital payment, online education, and live streaming.

#### 3.2.4. Data Collection

Questionnaire interviews were conducted during April–May 2022 in five elderly communities and two nursing homes in urban and rural areas of Nanjing, China. Disinfection and quarantine were conducted before and after the interviews, and thermometers and hand-washing were used. Liquids were provided to check the quarantine status of objects from time to time. Finally, 400 valid samples were obtained.

#### 3.2.5. Data Analysis

ATLAS.ti specializes in working with qualitative analysis texts [58]. Since the questionnaire data in this study were stored in text form, ATLAS.ti was chosen as the analysis tool for the text data. Moreover, the questionnaire results were analyzed using the selective optimization with compensation (SOC) model.

## 4. Results

### 4.1. Quantitative Results

#### 4.1.1. Descriptive Statistics of the Large-Scale Sample

Table 6 shows the descriptive statistics for the large-scale sample variables in the Yangtze River Delta region. Most of the older adults lived in urban areas, and a small proportion of them living in rural areas. In terms of age, the average age of the elderly was about 68 years old, and the number of males was slightly higher than the number of females. In terms of pension, a higher percentage of urban older adults had the pension compared to rural older adults. In terms of daily care and financial support, the majority of older adults did not rely on their children for financial support or care support. Rural older adults were less willing to participate in group activities than urban older adults and were less adept at managing interpersonal relationships. Urban older adults had greater access to aging-friendly facilities. The relationship between these variables and the quality of life for older adults is discussed in more detail below. 

#### 4.1.2. Impact of Digital Consumption on the Quality of Life for Older Adults

**Figure 7:**
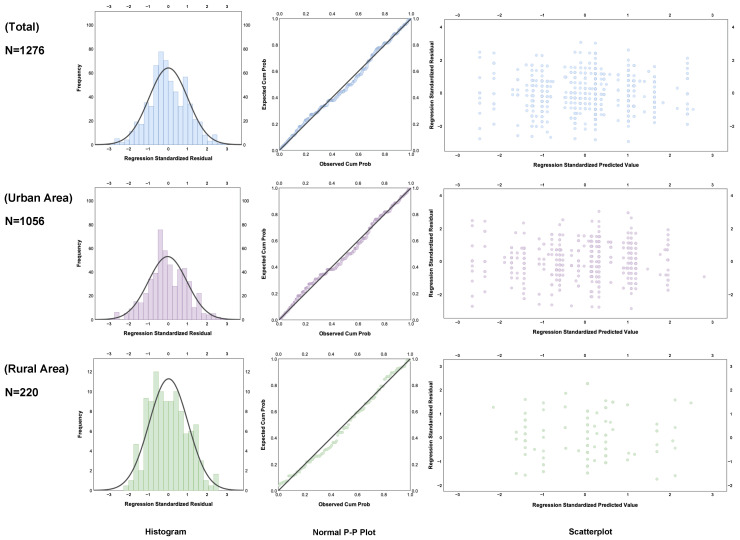
Residual analysis histogram with normal distribution.

In the regression model, the variables were structured as follows: one dependent variable was quality of life (QoL), three independent variables were digital life services (DC1), digital life affairs (DC2) and digital life entertainment (DC3), and six control variables were pension (PO), financial support from children (FS), group activities (GA), interpersonal relationships (RI), care support (CS), and facility convenience (FC). The results of the OLS regression analysis are shown in Table 7. As shown in Figure 7, the OLS regression results were subjected to residual analysis to check the fit of the model.

The R² of the three regression models was close to 1, which indicates a good fit, as shown in Table 7. The results of the residual analysis of the three regression models are displayed in Figure 7, which shows that the fit of the regression models in this study passed the residual test according to the normal distribution of the standard residual values with the graphical trend of the p–p plot.

According to the results shown in Table 7, the digital consumption variables DC1, DC2, and DC3 had a significant positive effect on QoL (*p* < 0.1) for the full sample and a more significant effect on older adults in rural areas. PO and FC had a significant positive effect on QoL (*p* < 0.05) and a greater degree of significant effect on older adults in rural areas. FS and CS both had a significant positive effect on QoL (*p* < 0.1) for the full sample and older adults in rural areas were more significantly affected by FS. GA and IR had a positive effect on QoL (*p* < 0.1) for the full sample, but not to a significant degree. 

To address the possible omission of control variables and the model setting limitations of the OLS model, this section used the PSM method for analysis as a reference for robustness testing of the OLS model. The matching method used was nearest neighbor matching, and the sampling method was put-back sampling.

First, a sample matching mass balance test was conducted, and the results are shown in Table 8. The matching results showed that the standardized error was significantly reduced after matching, which indicates that the matching effect was better. Moreover, the *t*-test before matching for some variables was significant (*p* < 0.05), but the *t*-test after matching was not significant (*p* > 0.05), which further indicates a better matching effect.

Next, to investigate whether there was a significant difference in the values of the outcome variables after successful matching, an ATT mean treatment effect analysis was conducted. The results are shown in Table 9, where the mean treatment effect value after matching was 0.16, indicating that older adults who had digital consumption behavior had 12.2% higher quality of life than the others.

### 4.2. Qualitative Results

#### 4.2.1. Descriptive Statistics of the Small-Scale Sample

An open-ended questionnaire interview was conducted with 400 elderly respondents in a small-scale sample in Nanjing, and the text description of the questionnaire is shown in Table 3. The average age of the small-scale sample was 67.6 years old. Half of the older adults had at least a junior high school education and were retired. A quarter of the older adults lived alone, and most lived with their partners or children.

The purpose of the open-ended questionnaire was to analyze how older adults coped with digital living services (DC1), digital living affairs (DC2), and digital living entertainment (DC3) during the pandemic. The small-scale sample was combed through ATLAS.ti, and the semantic network of the text was built by combining the selective optimization with the compensation (SOC) model. 

#### 4.2.2. Semantic Network on Digital Consumption for Older Adults

The concept of the SOC model consists of three main components, namely selection, optimization, and compensation. This section focuses on coding and classifying the results of the open-ended questionnaire according to the selection strategy, optimization strategy, and compensation strategy. 

For the construction of selection strategy coding, we defined the reasons for older adults to participate in digital consumption as selection strategies. For example, the expressions of older adults’ experiences with digital life services (DC1), digital life affairs (DC2), or digital life entertainment (DC3) related ground services would be categorized into selection strategies for digital consumption. 

For the construction of the optimization strategy coding, we defined the challenges for older adults in digital consumption with their expectations of digital consumption as the optimization strategy. For example, the older adults wanted to learn how to play a musical instrument on the Internet, but it required the older adults to overcome the hand–ear and screen delays, and this contradictory role of the relationship would be categorized into the optimization strategy of digital consumption. 

For the construction of the compensation strategy coding, we defined the representation of usability issues in digital consumption as the compensation strategy. For example, an older adult’s experience of pain relief through telemedicine would be categorized as an compensation strategy for digital consumption. 

**Figure 8:**
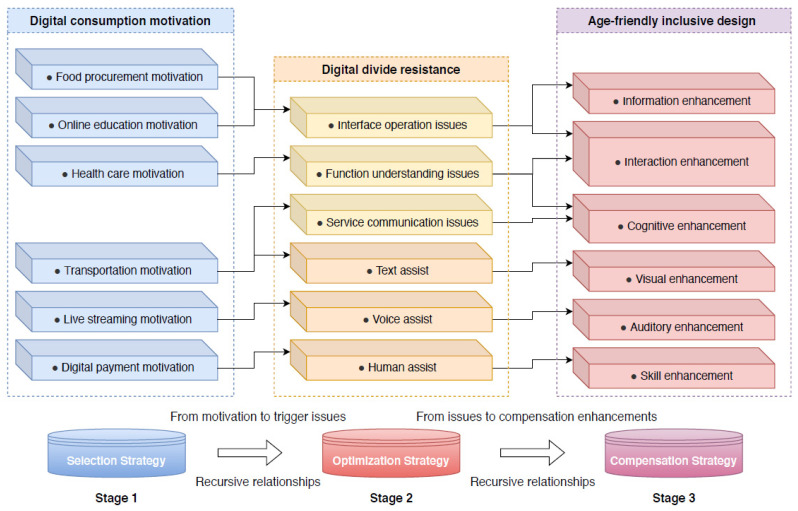
Semantic network relations of the digital consumption of older adults in a small-scale regional sample.

Based on the above construction idea, the semantic network relationship of digital consumption for older adults is shown in Figure 8.

#### 4.2.3. Analysis of Digital Consumption Strategies for Older Adults under the SOC Model

The questionnaire statements of older adults showed that older adults encountered barriers to the digital divide in food procurement, digital payments, and transportation, which led to negative comments. Older adults of different ages and regions did not show significant differences in their evaluation of strategies.

(1) Selection strategy in digital consumption

As shown in Figure 8, the selection strategies for older adults were mainly based on motivational factors, and the following different types of consumption forms provided more choices for older adults in the small sample in Nanjing such as food procurement motivation, online education motivation, health care motivation, transportation motivation, live streaming motivation, and digital payment motivation. At first, with many of the older adults in the open-ended questionnaire feedback, they were willing to continue to participate in digital consumption. For example, one older adult described the rationale for why they chose digital services for food procurement:

“Although the range of movement is limited during a pandemic, we can use the online community system to get food provided by the government within one kilometer. We enjoy the better service at the same price.”

(64, male, urban)

Several other older adults similarly described their food procurement experiences. Notable among these was the great sensitivity of older adults to the price factor. This is consistent with the positive effect of pensions on the quality of life in the large-scale sample.

Second, in the open-ended questionnaire, older adults also provided feedback on negative evaluations of digital consumption. The negative evaluations mainly stemmed from digital consumption media tools, and older adults preferred to use familiar digital products with interface styles. New interfaces and new interaction logics imply more cognitive burden and learning costs. Older adults possess technology acceptance, but once digital consumption behavior habits are created, it is difficult to change:

“Since digitizing my medical records, I can document my diabetes process more visually and better help with treatment. Through a year of use, I have become very proficient in using medical mobile apps. But I am very concerned about the app’s failure because I have not tried other companies’ app offerings.”

(60, female, rural)

Thus, the selection strategy as the first stage strategy in the digital consumption behavior performed by older adults is the overall criterion for older adults to consider the cost and process of digital consumption. Engaging or not engaging in digital consumption may seem like a split-second decision, but it contains other logics that require further discussion and specific problematic manifestations of the older adults’ negative evaluations.

(2) Optimization strategy in digital consumption

Optimization strategies for older adults are mainly influenced by resistance factors. As shown in Figure 8, the resistance to digital consumption among older adults comes from the following six categories of problems. These are interface operation issues, function understanding issues, service communication issues, text assist, voice assist, and human assist. Different degrees of physical and psychological aging bring different degrees of resistance to digital consumption. Interface operation, functional understanding, and service communication of digital products are the digital divides most frequently mentioned by older adults. The digital divide not only creates resistance to the external consumption behavior of the elderly, but also affects the internal consumption psychology of the elderly. There were many such feedbacks in the open-ended questionnaire:

“Presbyopia is so severe that I need to look at my cell phone far away from my eyes, but the distance is too far to operate it. When shopping online, too many items often make myself feel disoriented and dizzy.”

(65, female, urban)

While there were many negative comments from the digital divide, there was still positive expectation feedback such as:

“When using map navigation, I often can’t distinguish directions in a short period of time. Since I use a navigation app with a vibration feature, whenever I need to turn at an intersection, I can receive the vibration feedback to help me turn in time. I hope that such vibration cues can be used in other scenarios.”

(62, male, urban)

Thus, the optimization strategy, as the second stage strategy in the digital consumption behavior performed by the elderly, is a proactive or reactive demand solution to the manifestation of the resistance problem. The ability to solve the resistance problem requires an in-depth discussion on the way to strengthen the capabilities of the elderly.

(3) Compensation strategy in digital consumption

The compensation strategy for older adults is an aging-appropriate strategy that targets improvements and enhancements in aging functions to help older adults close the gap in the digital divide. As shown in Figure 8, older adults expected to improve their digital consumption experience in the following six areas: information enhancement, interaction enhancement, cognitive enhancement, visual enhancement, auditory enhancement, and skill enhancement. The compensation method that received the most feedback in the open-ended questionnaire was the mutual compensation of different sensory channels, thus helping older adults access information:

“The phone screen is too small and when learning online skills, I often use the auto-read aloud text feature to help me acquire knowledge by ear. Also, I don’t need to keep an eye on home security; the security system will sound an alarm when people walk through.”

(70, male, urban)

Compensatory strategies work not only with the older adults’ external information acquisition, but also with their internal knowledge acquisition. This is how some older adults described the process of enhancing their knowledge systems:

“Before I was exposed to digital services, I never owned a piano to learn to play because of the cost of space. But now I can play the piano just through online education and electronic simulators, and I can even learn to play any of the playing instruments.”

(65, female, urban)

Thus, the compensation strategy serves as the final stage strategy in the digital consumption behavior performed by older adults. The biggest difference compared to the choice and optimization strategies was that older adults made fewer active judgments and increased their inclusiveness of physical and virtual environments. Accepting the reality of aging body functions and finding a new niche in digital consumption is a manifestation of successful aging.

## 5. Discussion

The purpose of this study was to examine the effect of digital consumption on the quality of life of older adults as well as explore the variability of this impact effect in urban areas and rural areas. In addition, we assessed the digital consumption coping strategies of older adults during the pandemic.

To achieve the study objectives, we proposed an innovative research method of regional demographic characteristics and subjective evaluation for quality of life. The research method consists of a quantitative study with the large-scale sample and a qualitative study with the small-scale sample. The method aims to address the personnel consumption and time cost in quality of life evaluation research, especially in countries with a wide geospatial span. Multiple dimensional variables are taken for quantitative analysis studies for large-scale regional samples. At the same time, the selective optimization with the compensation (SOC) model was applied in qualitative analysis studies for small-scale regional samples. Among the results of this study, several findings are noteworthy in comparison with the existing studies.

### 5.1. Comparison of Research Findings 

First, research in the large-scale regional sample found that older adults in rural areas are more likely to be affected by digital consumption, which changes their quality of life. This finding can be explained by the existing aspiration level theory, which indicates that the lower living conditions and expectations, the more likely they are to improve the evaluation for their quality of life [59]. Additionally, male older adults had more subjective willingness to engage in digital consumption. Padilla-Meléndez et al. [60] and Assaker et al. [61] also confirmed the gender variability in the perceived usefulness of technology. In addition, the average quality of life assessment scores of older adults in rural areas were higher than those of older adults in urban areas. This may be due to the changing family and labor force structure, which in turn has led to a general lack of care for the elderly population among the youth group, contributing to the more stable quality of life of rural households [62]. By comparing the results with those of previous studies, it can be found that the results of the present study regarding the factors influencing quality of life are similar to those of existing studies. To a certain extent, it shows the feasibility and replicability of the research method in this study.

Second, given that there are few studies on the regional attributes of quality of life for older adults in China. This study attempted to explore the urban and rural variability in the quality of life for older adults. The findings obtained from the large-scale quantitative research sample showed that digital consumption had a significant positive correlation on the quality of life for older adults in different regions. The economic income factor showed a significant positive effect on the quality of life for older adults in different regions. This suggests that the economic power of older adults is the direct driver of digital consumption and may be influenced by other dimensions such as policy and technology. No economic power was found that could be influenced by other dimensions to change the consumption behavior. This finding is inconsistent with the argument that the forms and drivers of consumption can be altered by new regulations and procedures [63].

Third, financial and care giving support from children, according to the quantitative study data, did not have a significant impact on the quality of life of older adults in relation to digital consumption. This phenomenon can be explained by group moral theory, which holds that the traditional elderly moral ethic still exists in the elderly group, but changes in external environmental factors have led to a shift in the elderly ethic [64]. It also shows that there is a tendency among Chinese elderly groups toward a Western influenced ideology, and they are becoming more independent.

Finally, the small-scale qualitative sample showed that the digital divide and technology acceptance were associated with negative ratings of the older adults’ digital consumption response strategies. Specifically, cognitive load, tolerance, and technology acceptance were more likely to create barriers to the digital consumption behaviors of older adults. This finding is consistent with the research by Giansanti and Veltro [65], which suggests that the digital divide is the greatest usability barrier for older adults between the general consumption domain and the digital consumption domain.

### 5.2. Limitations and Future Research

This study also has certain limitations. First, the study only delineated three dimensions of behavior regarding digital consumption; therefore, the generalizability of these findings may be limited. Future research should combine the consumption business stage and consumer life course stage dimensions to expand the influencing factors.

Second, the propensity score matching method was applied in this study to the treatment effect of digital consumption on the quality of life. Because PSM matches are determined according to propensity scores, unsuccessful matching will delete part of the sample. Therefore, this approach requires a large sample size. Therefore, the generalizability of this method is not good for small regions and small samples. Future research should make use of different analysis methods to accommodate differences in sample sizes to establish a sound regional evaluation model of the quality of life.

Finally, this study was limited by the regional action of the pandemic. The qualitative analysis in this study was conducted only in Nanjing, China, and the sample of the change area was dominated by respondents with higher material and educational levels, and the overall development level of Nanjing may be slightly higher than the average; therefore, the qualitative findings may not be applicable in extremely economically undeveloped areas. Meanwhile, the qualitative questionnaire in this study was dominated by descriptive questions and neglected to examine the implicit needs of the respondents. Future research should consider more disaggregated regional variation factors, establish criteria for classifying regional differences, and incorporate research methods designed to reflect the implicit needs of the older population.

## 6. Conclusions

This study applied digital consumption variables to analyze the quality of life for older adults in different regions and an innovative mixed research method applicable to the elderly group was proposed. This contributes to the localization, regionalization, and differentiation of digital elderly service development. Although quantitative data studies cover a wide range of areas, they often ignore the huge geographical scope of China, and the geographical differences give rise to regional cultural, regional demographic characteristics, and regional environmental differences. The subjective evaluations of the elderly population in this study are summarized to compensate for the variability generated by geographic distance. Face-to-face service thinking in virtual space will be a future trend in the digital senior care industry. For example, many digital consumer applications in China differentiate between provincial and urban areas to provide more accurate services to clients [66]. In the following, the main contributions of this study at the theoretical and practical levels are detailed.

### 6.1. Theoretical Implications

In terms of the theoretical implications, this study makes a partial contribution to the literature. First, in this study, we defined the scope of digital consumption from the perspective of the community service needs of older adults, bridging the gap between the older adults’ digital skills, digital interactions, and older adults’ needs perspectives. Existing indicators of quality of life for older adults have focused on Western societies, and quality of life is easily influenced by economic and geographical factors. Therefore, appropriate life evaluation improvement criteria should be developed for countries with strong economic and geographic characteristics.

Furthermore, this study on digital consumption is one of the few regional studies conducted on economics and gerontology in China, specifically during an epidemic. After reviewing the literature and conducting an empirical study, a significant positive correlation of digital consumption with the quality of life of older adults in different regions was found. The digital divide was also found to produce negative subjective evaluations of the digital consumption response strategies of older adults. This finding helps to model the shift from the traditional consumption system to the digital consumption system from a geographical perspective.

### 6.2. Practical Implications

China’s growing elderly population has created a huge demand for senior care services, and all industries are undergoing digital transformation. The number of Internet users among China’s aging population is growing much faster than other age groups, and how to develop and increase consumption in the digital economy is a key concern for all aging countries and regions. Notably, this study provides a reference for dealing with the relationship between older people and digitalization. In particular, the cost of population research is considered, and lower operating costs will make the digital elderly service industry more competitive.

## Figures and Tables

**Table 1:** Classification of studies on the quality of life in older age groups during the pandemic.

Authors	Type	Contest of Application	Territorial Scale	Summary
Yildirim et al. (2021) [47]	Negative	Malatya, Turkey	Regional	During the pandemic, older adults become miserable, tired, and depressed. This leads to a rise in their negative emotions.
Guida and Carpentieri (2021) [43]	Negative	Milan, Italy	Urban	Measuring the quality of life of the older adults affected by the coronavirus in Milan in common work scenarios and during the pandemic.
Colucci et al. (2022) [42]	Negative	Quebec, Canada	Regional	The blockade during the pandemic led to a decline in the quality of life and well-being of the elderly.
Harrison et al. (2021) [48]	Positive	Washington, DC, US	Urban	Minority and non-minority physical activity has decreased since COVID-19, but no change in quality of life was found.
Lee and Hsu (2021) [44]	Positive	Taiwan	Regional	The skills of seniors are enhanced through in-home education, thus giving them a new experience.
Ammar et al. (2021) [49]	Positive	Magdeburg, German	Regional	Older adults used ICT solutions to improve confidence in health monitoring, improving their quality of life.
Sheykhangafshe et al. (2021) [45]	Positive	Tehran, Iran	Urban	Older adults in religious affiliations were more resilient during the pandemic, and more tolerant of the resulting stress and pain, which in turn improved their quality of life.
Duan et al. (2021) [50]	Positive	Wuhan, China	Regional	Importance of enhanced vegetable intake and preventive behaviors to improve the quality of life in older adults during the COVID-19 pandemic.

**Table 2:** Measurements of the dependent variable quality of life (QoL).

Variables	Measurement
GH	Respondents’ self-evaluation of general health. (Five-point scale: 1 = Very weak, 5 = Very healthy)
PF	Respondents’ self-evaluation of physical function. (Five-point scale: 1 = Very disabled, 5 = Very complete)
RP	Respondents’ self-evaluation of role physical. (Five-point scale: 1 = Very slow, 5 = Very fast)
BP	Respondents’ self-evaluation of body pain. (Five-point scale: 1 = Very often, 5 = Not at all)
VT	Respondents’ self-evaluation of vitality. (Five-point scale: 1 = Very lethargic, 5 = Very active)
SF	Respondents’ self-evaluation of social function. (Five-point scale: 1 = Very introverted, 5 = Very outgoing)
RE	Respondents’ self-evaluation of role emotion. (Five-point scale: 1 = Very depressed, 5 = Very pleased)
MH	Respondents’ self-evaluation of mental health. (Five-point scale: 1 = Very weak, 5 = Very healthy)

Note: GH—general health; PF—physical function; RP—role physical; BP—body pain; VT—vitality; SF—social function; RE—role emotion; MH—mental health.

**Table 3:** Measurements of the independent variable digital consumption (DC).

Variables	Measurement
DC1	Have you ever used digital consumption for life services? (Dichotomous variables: 0 = No, 1 = Yes)
DC2	Have you ever used digital consumption for life affairs? (Dichotomous variables: 0 = No, 1 = Yes)
DC3	Have you ever used digital consumption for life entertainment? (Dichotomous variables: 0 = No, 1 = Yes)

Note: DC1—digital life services; DC2—digital life affairs; DC3—digital life entertainment.

**Table 4:** Measurements of the control variables.

Control Variables	Measurement
PO	Do you have a pension? (Dichotomous variables: 0 = No, 1 = Yes)
FS	Do you have financial support from your children? (Dichotomous variables: 0 = No, 1 = Yes)
GA	Are you willing to participate in group activities? (Dichotomous variables: 0 = No, 1 = Yes)
IR	Are you good at handling relationships with others? (Dichotomous variables: 0 = No, 1 = Yes)
CS	Do you have care support from your children? (Dichotomous variables: 0 = No, 1 = Yes)
FC	Are there aging-friendly facilities close to home? (Dichotomous variables: 0 = No, 1 = Yes)

Note: PO—pension; FS—financial support for children; GA—group activities; IR—interpersonal relations; CS—care support; FC—facility convenience.

**Table 5:** The open-ended questionnaire description.

Categories	Questionnaire Description
DC1	
1. Food procurement	Have you ever chosen to get food online? Please describe the experience of using it.
2. Health care	Have you ever remote diagnosis and access to medical records? Please describe the experience of using it.
DC2	
3. Transportation	Have you ever used the electronic reservation system for transportation? Please describe the experience of using it.
4. Digital payment	Have you ever used paperless trading? Please describe the experience of using it.
DC3	
5. Online education	Have you ever used the Internet to learn a skill? Please describe the experience of using it.
6. Live streaming	Have you ever tried to live-stream your life in the network? Please describe the experience of using it.

Note: DC1—digital life services; DC2—digital life affairs; DC3—digital life entertainment.

**Table 6:** Descriptive statistics of the characteristic variables of older adults.

Categories	Variables	Total (N = 1276)		Urban Area (N = 1056)		Rural Area (N = 220)	
		Mean Value	Standard Deviation	Mean Value	Standard Deviation	Mean Value	Standard Deviation
Dependent variable	QoL	26.92	3.34	26.88	3.30	27.13	3.55
Independent variable	DC	0.61	0.98	0.70	1.03	0.17	0.52
Control variable	PO	0.66	0.48	0.66	0.47	0.64	0.48
	FS	0.15	0.36	0.15	0.36	0.15	0.36
	GA	0.70	0.46	0.71	0.45	0.63	0.49
	IR	0.31	0.46	0.35	0.48	0.10	0.30
	CS	0.13	0.30	0.14	0.29	0.12	0.21
	FC	0.68	0.46	0.70	0.46	0.60	0.49
Demographic variable	Gender	0.52	0.50	0.52	0.50	0.53	0.50
	Age	68.12	5.30	68.04	5.43	68.48	4.64

Note: QoL—quality of life; DC—digital consumption; PO—pension; FS—financial support for children; GA—group activities; IR—interpersonal relations; CS—care support; FC—facility convenience.

**Table 7:** OLS regression results on the quality of life for older adults.

Variables	Total (N = 1276)			Urban Area (N = 1056)			Rural Area (N = 220)		
	Standard Errors	*p*	VIF	Standard Errors	*p*	VIF	Standard Errors	*p*	VIF
DC1	0.49	0.095 *	1.48	0.59	0.097 *	1.50	0.62	0.093 *	1.50
DC2	0.21	0.085 *	1.12	0.19	0.088 *	1.11	0.80	0.082 *	1.25
DC3	0.46	0.091 *	1.77	0.48	0.095 *	1.73	0.84	0.087 *	2.11
PO	0.28	0.034 **	1.00	0.31	0.050 *	1.00	0.70	0.017 **	1.05
FS	0.37	0.069 *	1.03	0.41	0.093 *	1.04	0.92	0.044 **	1.03
GA	0.62	0.093 *	2.53	0.70	0.090 *	2.59	1.38	0.095 *	1.48
IR	0.21	0.078 *	2.82	0.23	0.066 *	2.89	0.80	0.078 *	2.15
CS	0.34	0.069 *	1.33	0.33	0.069 *	1.32	0.85	0.069 *	2.31
FC	0.61	0.035 **	2.68	0.69	0.034 **	2.81	1.37	0.036 **	2.49
R^2^	0.72			0.66			0.78		
F	F = 5.62 *p* = 0.027 ** 5.30			F = 4.78 *p* = 0.032 ** 5.43			F = 6.04 *p* = 0.022 ** 4.64		

Note: DC1—digital life services; DC2—digital life affairs; DC3—digital life entertainment; PO—pension; FS—financial support for children; GA—group activities; IR—interpersonal relations; CS—care support; FC—facility convenience. * means *p* < 0.1, ** means *p* < 0.05.

**Table 8:** The matching mass balance test.

Variables	Status	Treated (N = 220) Control	Control (N = 220)	*p*	Standard Error (%)	Difference (%)
		Mean	Mean			
DC1	Unmatched	0.66	0.45	0.04 **	5.25	81.25
	Matched	0.65	0.57	0.37	2.00	
DC1	Unmatched	0.34	0.15	0.14	4.75	18.75
	Matched	0.36	0.20	0.70	4.00	
DC1	Unmatched	0.20	0.15	0.25	1.25	12.50
	Matched	0.20	0.17	0.89	0.75	
PO	Unmatched	0.62	0.58	0.02 **	1.00	18.75
	Matched	0.61	0.60	0.67	0.25	
FS	Unmatched	0.44	0.31	0.04 **	3.25	31.25
	Matched	0.44	0.36	0.83	2.00	
GA	Unmatched	0.71	0.63	0.67	2.00	12.50
	Matched	0.72	0.66	0.65	1.50	
IR	Unmatched	0.55	0.48	0.02 **	1.75	18.75
	Matched	0.55	0.51	0.65	1.00	
CS	Unmatched	0.16	0.14	0.26	0.50	6.25
	Matched	0.15	0.14	0.52	0.25	
FC	Unmatched	0.85	0.81	0.65	1.00	12.50
	Matched	0.85	0.87	0.67	0.50	

Note: DC1—digital life services; DC2—digital life affairs; DC3—digital life entertainment; PO—pension; FS—financial support for children; GA—group activities; IR—interpersonal relations; CS—care support; FC—facility convenience. ** means *p* < 0.05.

**Table 9:** Propensity score match estimation results.

Matching Method	Treated (N = 220)	Control (N = 220)	ATT	*p*
Unmatched	27.13	27.25	0.12	0.04 **
Nearest neighbor matching	27.36	27.50	0.16	0.02 **

Note: ** Means *p* < 0.05.

## Data Availability

Not applicable.
